# The Public Health: Interviews with Local Authorities

**Published:** 1922-07

**Authors:** 


### THE PUBLIC HEALTH.

### INTERVIEWS WITH LOCAL AUTHORITIES.

### I.—LIVERPOOL.

IN a corner of the splendid building in which the museum of the Liverpool University School of Hygiene is housed, is a quaint print surmounted by a wavy piece of ribbon, borne aloft by cupids, and bearing the superscription "Liverpool in Lancashire." The place so depicted—and the period is as recent as the middle of the eighteenth century—appears to have been a mere handful of houses with a church or two standing up above a rippling stream, and with fields and windmills in the immediate vicinity. Underneath this idyllic picture we read "This Corporation is governed by a Mayor and Aldermen whose reputation is augmented by many Gent of the considerablest families in the County. This is the most flourishing and populous seaport town in these parts, being beautiful in its buildings, and no less pleasant in a large and well-contrived Dock, wherein ships are laden and unladen before the Custom House."

Liverpool was the first borough in the county to have a Medical Officer of Health—in the year 1846—and two or three entries taken from the annals of Liverpool in the following two years show that the Health Committee and their Medical Officer were likely to have their powers of administration severely taxed. Thus—

1847.—Fever sheds, Mount Pleasant, opened April 15; about 15,000 deaths occurred by fever and famine.

1847.—Health Committee caused during this year 14,085 cellars to be measured and registered, of which number 5,841 contained wells of stagnant water beneath the floors. Notices to quit were served upon 8,878 of the occupants of these miserable abodes; 5,000 of these were wholly cleared of the inmates.

1848.—Great influx of Irish paupers—no less than 296,231 immigrated into the town during twelve months ended July 30.

Even allowing for artistic licence in the middle of the eighteenth century, it is evident that Liverpool, in the short space of 80 or 90 years, had rapidly deteriorated in matters of health and pleasant environment, as her industries had developed. And the field of activity for the Health Authorities of to-day was considerably widened during the remaining half of the nineteenth century. To those authorities, the successors of the gentlemen of augmented reputation of the eighteenth century, we went to learn something of what Liverpool is now doing and thinking in regard to matters of health. We were most courteously received by Mr. Hughes, the Deputy Chairman of the Health Committee (in the absence of Alderman Muirhead, the Chairman), and by Dr. Hope, the Medical Officer of Health, who—with other members of the medical staff—most obligingly assisted us in every way with our inquiries. It is not possible within the reasonable limits of a single interview to do justice to the activities of the Liverpool Health Committee and their Medical Officer but we have set down in the following paragraphs some of Mr. Hughes' and Dr. Hope's replies.

#### Infectious Diseases.

If in Liverpool, they said, we have an outstanding responsibility not only to ourselves but to the country generally, it is in regard to the control of infectious disease. The large volume of shipping arriving from all parts of the world impose on us the need for constant vigilance and the closest application of all measures having for their object the prevention of all forms of infectious disease. The last published figures of deaths—those for 1920—bear eloquent witness to the success of these measures—

| | No. of Deaths. | Maximum Number recorded for any previous year. |
|---|---|---|
| Smallpox .. | 2 | .. 1,919 |
| Typhus .. | Nil | .. 1,474 |
| Cholera .. | Nil | .. 1,782 |
| Enteric fever | 8 | .. 248 |
| Scarlet fever | 70 | .. 1,911 |

*Rats.*—Special attention is given to the bacteriological examination of rats, and to measures for their extermination. Ships moored to the quays are required to have their mooring ropes fitted with metal shields, designed to prevent the rats from landing by coming down the mooring ropes. Persons are specially employed as rat-searchers and as rat-catchers, and the co-operation of owners of buildings sought in the clearance of all accumulations of refuse, as far as possible by burning, and in other ways. Systematic periodical surveys are conducted in the City in connection with the campaign against rats.

*The Minor Infectious Diseases.*—We are seeking fresh Parliamentary powers for strengthening our hands in applying preventive measures against measles and whooping cough. These are, unfortunately, too often regarded as "trifling" forms of illness, and we therefore direct attention to the fact that they have destroyed in recent years in Liverpool more than twice as many lives as all of the other forms of infectious disease, excluding phthisis, put together.

#### Venereal Disease—Failure of Present Measures.

We have our special difficulties in Liverpool in this matter with the constant influx of foreign seafarers. We do not hesitate to express our view that the failure of the present system is manifest, and that sterner measures are necessary. This disease, in addition to being most destructive to life and health, shares with no other infectious disease—excepting perhaps smallpox, and there only to a very limited extent—the characteristic of being transmissible to a future generation The treatment centres are well attended, but the patients leave as soon as the relief of actual physical suffering is attained. We believe our system of clinics in Liverpool to be as good as any in the Kingdom, and more advanced than most, but 60 per cent. of the patients cease to attend long before they are free from infection. If such a state of affairs is visualised for a moment in regard to smallpox or scarlet fever, the absurdity of expecting any success in the direction of *prevention* will be manifest.

If powers of compulsion in this matter are to be regarded as experimental in the first instance, well and good; but we think that Liverpool is a most suitable place for putting into force experimental powers. We are not prepared, if we can help it, to wait while a health resort, for example, with no workshops, no seafaring population—no serious health problems of any kind—concerns itself with this, to us, pressing question. We are, therefore, seeking Parliamentary powers designed, broadly speaking, to ensure :—

(i.) Compulsory Notification ; and
(ii.) Compulsory Treatment, *to be continued* until there is freedom from infection.

### Death-Rate.

In the last published report (for 1920) we record the lowest death-rate, 16·4 per 1,000, ever recorded in the City; the report, however, for 1921, about to be published, shows an improvement on this, with a death-rate of 14·3. Our average death-rate per 1,000 of the population has been :—

| | | |
|---|---|---|
| For the period | 1881-1890 | 26·1 |
| „ „ „ | 1891-1900 | 23·9 |
| „ „ „ | 1901-1910 | 20·0 |
| „ „ „ | 1911-1920 | 18·1 |

Notwithstanding that so much remains to be done, encouragement—but not complacency—is to be derived from these comparative statistics.

It is not practicable in one interview to deal adequately with two very substantial questions—Housing and Tuberculosis—which have an important bearing on the improvement shown by these figures and the further improvement which, we hope, will be effected in the future.

### Housing.

With regard to housing, we have put up 4,000 houses since the War and are making steady progress with the clearance of slum areas. Of course, this is not to say that a great amount does not remain to be done, and that progress has become increasingly difficult owing to the Government's alteration in their housing programme. As an illustration of what remains to be done in regard to slum clearance, it may be pointed out that some 2,500 houses, mainly of the back-to-back type, in unhealthy areas have not yet been dealt with, and the death-rate in these areas varies from 20 to nearly 50 per thousand—the corresponding infant-mortality rate, per thousand births, varying from 112 to the appalling figure of 413.

### Tuberculosis.

With regard to tuberculosis, our activities in relation to the treatment of every form of tubercular disease are, we believe, second to none in the country, and we should be pleased, if an opportunity offers, to give full account of what we are doing in regard to all types of cases in the direction of institutional and dispensary treatment, nursing and after-care.

In considering the future welfare of Liverpool, our work—

(1) in preparing fresh workers in the field of health, and
(2) in looking after the children, is of paramount importance.

### The School of Hygiene.

We feel that we are justly proud of our School of Hygiene.* This is a joint effort on the part of the University and the Corporation at a cost of £30,000. In the same building are the lecture theatre, classrooms, and museum; the City Analyst and the City Bacteriologist carry on their work here, and the well-equipped laboratories are available both for the practical day-to-day work and for teaching facilities for the 100 students who attend the classes here. The school is always full. We claim to be doing good, sound, pioneer work here, and why the Minister of Health, by his recent Order, proposes in effect to deprive us of an important part of our functions by refusing to recognise diplomas issued to our students seeking posts as sanitary officers —while we are allowed to continue to issue such diplomas to medical graduates—we are at a loss to understand.

### Midwives.

With very few exceptions, the midwives of the City are fully trained. They are nearly all members of an association and, through this body, are closely linked up with sanitary administration, and themselves arrange special courses of instruction from the gynæcologists of the City.

### Maternity and Child Welfare.

We have encouraged and given every assistance to all forms of voluntary organisation helping in this direction, and, in districts where these arrangements have been insufficient, ante-natal centres and infant clinics have been established. We should like to emphasise the fact that in this work on the medical side we rely almost entirely on the general practitioners. Our arrangements are such as to bring all, or nearly all, mothers and infants and children up to the first four or five years of their lives under our special supervision. There were some 71,000 attendances at the infant clinics in 1920, and a similar number at the day nurseries, of which there are nine with accommodation for 390 children.

*An Administrative Blunder.*—It is an administrative blunder that the continuity of this supervision should be broken when the child reaches its fifth birthday, but we are glad to say that, in Liverpool, the Medical Officer of Health is also the Medical

* We were taken over this most interesting building by Dr. Mussen. It is well worth a separate article when opportunity offers.—Ed.

Officer of the Education Committee, and that some linking up of the activities of the two bodies is in this way rendered possible. But it would be of enormous advantage that the same Medical Officers and nurses dealing with the children from birth should carry on the supervision through school life. The greater confidence of the parents would be secured, the constant association of the same doctors and nurses would bring about a more friendly feeling, and they would come to regard the Medical Officer as a sort of family doctor.

*Maternity and Rest Home.*—This Home has proved to be of immense benefit to the patients (some 150 in number each year). These are women whose physical condition or home circumstances make it desirable that they should have rest and care before, during, or after their confinements. The Home was provided and equipped by the Maternity and Child Welfare Sub-Committee, aided by the generosity of the American Red Cross Society and the Stanley Rogers Memorial Committee.

### Special Features in Annual Reports.

We have found it of great advantage to have special features in our annual reports with pictures and diagrams—*e.g.*, for 1911 (an exceptionally hot summer) particular attention was directed to the relation between the temperature, the prevalence of flies, and the number of deaths from diarrhœa. (Diagrams published since show a close relation between the number of flies caught and the prevalence of diarrhœa.)

In another year, a special series of pictures showed insanitary cowsheds with hardly any light or ventilation, farmyards with accumulations of manure, and little or no drainage—from which tubercular milk was finding its way into Liverpool, until the supply was prohibited as a result of the Medical Officer's activities; and, by way of contrast, a well-lit, well-ventilated and clean interior.

In other years we gave most instructive photographs of old and insanitary dwellings, of dark and unsavoury courts and interiors, and, on the other hand, modern and healthy dwellings erected, slum areas in process of being cleared, and showing street widening.

Another example of photographic demonstration shows (*a*) a mother and father and family of ten sturdy children, and (*b*) a similarly arranged picture, but with nine tragic spaces, showing the almost entire extinction of another family due to insanitary environment.

These illustrations are, in our view, a valuable feature of the yearly reports, arousing interest and actively helping to enlighten and mould public opinion.

### Local Acts.

We are strong advocates of local Acts of Parliament. If a health measure appears to Liverpool to be essential, the health authorities of that city are not prepared to wait while the rest of the country is being educated, but rather are prepared to demonstrate, by the local operation of particular measures, the value of those measures.

To the foresight and persistence of Local Health Committees are due many Health Acts of Parliament now in operation for the whole country. The series of "Notification" Acts, provisions with regard to housing and town-planning, measures dealing with the mother and child; all these had their forerunners in the shape of local Acts, whether in Liverpool or elsewhere, which gave to movements the necessary impetus, demonstrated by experience the value of theories, and generally paved the way for the extension of beneficent measures to the whole country.

### The Help (or otherwise) of the Central Departments.

In Liverpool we are strong supporters in principle of a Ministry of Health. Where health and other interests have clashed in the past, departments have often been, not merely lukewarm, but actually antagonistic to health measures. By way of illustration we point to a matter already referred to as specially concerning the port of Liverpool. Knowing the grave menace of plague-infected rats finding their way ashore from infected ships, the Port Sanitary Authority at Liverpool sought powers from Parliament to prohibit the practice of rat-catchers bringing rats ashore. Yet this most necessary measure was strongly opposed by a Government Department—happily without success.

While our relations with the Ministry of Health are, we are pleased to think, cordial, we do not hesitate to say that any Ministerial system of detailed control of local administrative measures is an impossibility. The recommendation of the Geddes Committee that there should be block grants to local authorities, to be paid, presumably, on the central department being satisfied of a good all-round standard of performance—we being free to incur our expenditure in details without interference—points the way, in our view, to an excellent plan.

### One Authority for all Health Measures—Ultimately.

The soundness of the principle, that there should ultimately be one local health authority controlling or co-ordinating all forms of health activity—curative and preventive—in the area, cannot, we think, be questioned, but so much requires to be done on the Preventive side with material more readily to hand before seeking a wider field of energy.

*Meanwhile, Preventive Measures.* — Preventive measures do not make the same appeal to the imagination as do curative measures, and it is paradoxical, but true, that the more successful they are, the less apparent, to the general public, is the need for them. We shall continue to pursue our progressive policy with preventive measures designed to make Liverpool in each succeeding year a better, a healthier city, feeling confident that we shall continue to receive the necessary support, financial and otherwise, of our fellow-citizens so long as they are kept fully informed as to the need. We ourselves are under no illusions on the subject, notwithstanding that so much has already been accomplished.

